# Insulin use in chronic kidney disease and the risk of hypoglycemic events

**DOI:** 10.1186/s12882-022-02687-w

**Published:** 2022-02-21

**Authors:** Daulton Grube, Guo Wei, Robert Boucher, Nikita Abraham, Na Zhou, Victoria Gonce, Judy Carle, Debra L. Simmons, Srinivasan Beddhu

**Affiliations:** 1 University of Washington School of Medicine, Seattle, Washington USA; 2 Department of Internal Medicine, Division of Nephrology & Hypertension, University of Utah Health Sciences, Salt Lake City, UT USA; 3 Study Design and Biostatistics Center, University of Utah Health Sciences, Salt Lake City, UT USA; 4 Medical Service, Veterans Affairs Salt Lake City Health Care System, Salt Lake City, UT USA; 5 Department of Internal Medicine, Division of Endocrinology, University of Utah Health Sciences, Salt Lake City, UT USA; 6 University of Utah Health Sciences, 421 Wakara Way Suite 360, Salt Lake City, UT 84108 USA

**Keywords:** Insulin, CKD, Diabetes, Hypoglycemia

## Abstract

**Background:**

We examined in persons with type 2 diabetes (T2D) whether the use of insulin and the risk of serious hypoglycemic events with insulin is higher in persons with more advanced CKD.

**Methods:**

In a national cohort of 855,133 veterans with T2D seen at Veteran Affairs clinics between Jan 1, 2008 and December 31, 2010 with at least two serum creatinine measurements, we defined insulin use from pharmacy records and serious hypoglycemic events by ICD-9/10 codes from emergency room visits or hospitalizations that occurred until December 31, 2016.

**Results:**

Mean age was 66 ± 11 years and 97% were men. Mean baseline eGFR was 73 ± 22 ml/min/1.73 m^2^. In a multivariable Cox regression model of those without insulin use at baseline (*N* = 653,200), compared to eGFR ≥90 group, eGFR < 30 group had higher hazard (HR 1.80, 95% CI 1.74 to 1.88) of subsequent insulin use. In a multivariable Cox model with propensity score matching for baseline insulin use (*N* = 305,570), both insulin use (HR 2.34, 95% CI 2.24 to 2.44) and advanced CKD (HR 2.28, 95% CI 2.07 to 2.51 for comparison of eGFR < 30 to eGFR ≥90 ml/min/1.73 m^2^ groups) were associated with increased risk of subsequent serious hypoglycemic events.

**Conclusions and relevance:**

In T2D, more advanced CKD was associated with greater insulin use. Both insulin use and advanced CKD were risk factors for serious hypoglycemic events. The safety of insulin compared to newer glycemic agents in more advanced CKD needs further study.

**Supplementary Information:**

The online version contains supplementary material available at 10.1186/s12882-022-02687-w.

## Background

There are more than 30 million adults with diabetes mellitus (DM) in the United States [1]. Diabetes is the leading cause of chronic kidney disease (CKD) with almost 1 in 3 persons with diabetes developing kidney disease [2]. Despite the public health importance of kidney disease in persons with type 2 diabetes (T2D), there is a paucity of data on optimal treatment for glycemic control in this population [3]. Fundamental questions such as the role of insulin in glycemic control in CKD still need to be addressed.

It is often considered that insulin requirements go down with advanced CKD as insulin is cleared by the kidney [3–5]. However, cross-sectional studies suggest higher insulin use in persons with more advanced CKD [6–8]. Therefore, it remains unclear whether the need for insulin is decreased or increased in advanced CKD.

A serious adverse effect of insulin therapy is hypoglycemia that results in emergency room visit or hospitalization. While insulin therapy is a known risk factor for hypoglycemic episodes [10], whether advanced CKD by itself is associated with increased risk of hypoglycemia has been controversial. The incidence of hypoglycemia defined as blood glucose < 70 mg/dl was higher in those with more advanced CKD in a study of veterans [9]. However, a prospective observational study of individuals with T2D using continuous glucose monitors found that hypoglycemia was common in persons with moderate to severe CKD but was not more common than in those with preserved GFR [9]. The question of whether both insulin use and advanced CKD are independently associated with increased risk of hypoglycemia may have therapeutic implications for glycemic control in persons with T2D and CKD as hypoglycemic episodes are associated with increased risk of CKD progression [10], stroke [11] and mortality [12, 13].

Therefore, in the current study we examined the hypothesis that requirement for insulin for glycemic control in T2D is lower in those with advanced CKD. We also examined whether the incidence of serious hypoglycemic episodes is greater in those with more advanced CKD and augmented in those with more advanced CKD on insulin.

## Materials and methods

### Patient population

**Fig. 1:**
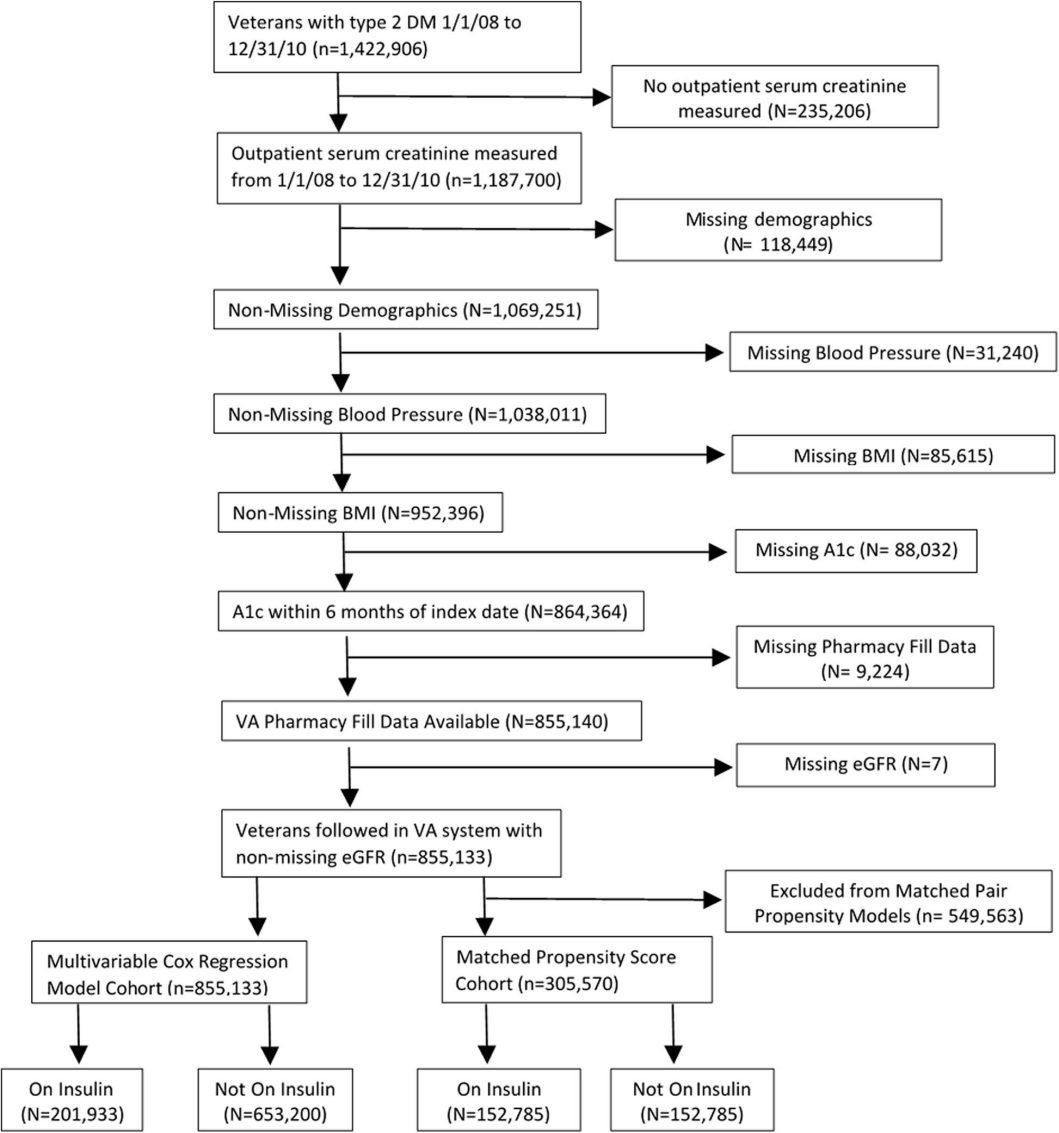
Flow diagram of study cohort

This was an observational study of veterans in the United States with an encounter in the Veteran Affairs (VA) system corresponding to a diagnosis of T2D between January 1, 2008 and December 31, 2010, and who had at least two outpatient serum creatinine measurements during the same time-period. The index date was defined as the date of the second outpatient serum creatinine value. Among the 1,187,700 veterans with T2DM and two outpatient serum creatinine measurements, the analytic cohort (*N* = 855,135) included those with non-missing data for demographics, blood pressure, body mass index (BMI) and hemoglobin A1C (HbA1c) and VA pharmacy prescription fill data (Fig. 1). This study was conducted with approval from the University of Utah Institutional Review Board as well as in accordance to the Declaration of Helsinki.

### Data source and covariates

We used the VA Informatics and Computing Infrastructure (VINCI) [14] platform to access the national VA data. Age (defined at index date), gender and race were obtained from the VA Corporate Data Warehouse (CDW) [15] data. Diagnosis of T2D was defined by International Classification of Disease (ICD-9) codes (Supplemental Table 1) recorded between 1/1/2008 to 12/31/2010 in VA Inpatient and Outpatient Medical SAS Datasets [16, 17]. While we required an ICD-9 diagnostic code of T2D mellitus to minimize the inclusion of type 1 diabetes mellitus in the study cohort, duration of T2D was calculated by the first occurrence of ICD-9 code for T2D, hemoglobin A1C > 6.5% or use of anti-diabetic medications from Oct 1, 1999 to the index date. Baseline comorbidities included coronary artery disease, congestive heart failure, cerebrovascular disease, peripheral vascular disease, lung disease and cancer. These were considered present if they were recorded in the VA Inpatient and Outpatient Medical SAS Datasets within 3 years prior to the index date. As the index date was defined by the second outpatient serum creatinine and the outpatient labs could be drawn either before or after a clinic visit, we used ±90 days window around the index date to capture clinic visits data such as blood pressures and weight. The first recorded outpatient systolic and diastolic blood pressures within 90 days of the index date were used. BMI was calculated using the first weight value recorded within 90 days of the index date and most recently recorded height value, not restricting to within the last 90 days. As HbA1c is measured only every 3 months (particularly in those not under good glycemic control) and even less frequently in clinical practice (particularly in those at glycemic control goal), we used ±180 days window around the index date to minimize missing HbA1c data.

Veterans’ prescription records [18] were used to identify baseline medication exposure of hypoglycemic agents, antihypertensive medications and statins. As many medications are commonly filled for 90 days, we applied ±90 days window around the index date to capture baseline medications. For baseline insulin use, we applied ±180 days window in order to minimize misclassification of new insulin use for those who might have missed filling their insulin in the 90 days window around the index date. Hypoglycemic agents were classified as insulin, sulfonylureas, metformin, thiazolidinedione (TZD) and other agents. Antihypertensive medications were classified as alpha-blockers, beta-blockers, calcium channel blockers, angiotensin converting enzyme inhibitors or angiotensin receptor blockers (ACEI/ARB), loop-diuretics and thiazide-type diuretics.

In the current analysis, we followed the veterans until death or the administrative censor date of December 31, 2016.

### Prevalent and new insulin use

Prevalent insulin use was identified through review of the patient’s prescription data within 180 days of the index date. In those not on insulin at baseline, incidence of new insulin use was determined by the first time that the patient had a prescription for insulin after the index date.

### Hypoglycemic episodes

We defined serious hypoglycemic events as those that needed medical attention as evidenced by an emergency room visit with a diagnosis of hypoglycemia and/or hospitalization with a primary discharge diagnosis of hypoglycemia codes. A previously validated definition [19, 20] was used to identify hypoglycemic events from ICD 9/10 codes from emergency room visits or hospitalizations that occurred from the index date until the censor date. (Supplemental Table 2).

#### Statistical analyses

Baseline characteristics of participants with and without insulin use were described by means and standard deviations or medians and interquartile ranges for numeric variables, and proportions for categorical variables.

#### Associations of eGFR categories with baseline and subsequent insulin use

In the entire analytic cohort (*N* = 855,133), we used a multivariable logistic regression model to relate baseline insulin use as the dependent variable to eGFR groups as the predictor variable, with eGFR ≥90 ml/min/1.73 m^2^ as the reference group. This model included adjustment for age, gender, race, coronary artery disease, stroke, peripheral vascular disease, congestive heart failure, lung disease, cancer, systolic and diastolic blood pressures, BMI, HbA1c, diabetes duration, use of ACE-Is or ARBs, sulfonylurea, metformin, TZDs, and other hypoglycemic agents.

In those not on insulin at baseline (*N* = 653,200), we used a multivariable Cox regression to estimate the association of subsequent, new insulin use by different eGFR groups with eGFR ≥90 ml/min/1.73 m^2^ as the reference. This model was adjusted for the same variables as above.

#### Associations of insulin use and eGFR categories with serious hypoglycemic events

To examine whether insulin use and eGFR categories were independently associated with serious hypoglycemic events, we first conducted a multivariable Cox regression analysis in the entire analytic cohort (*N* = 855,133) with the same covariates as above. To further reduce the risk of indication bias between veterans not on and on insulin use at baseline, we repeated the multivariable Cox regression model for serious hypoglycemia events in a propensity score matched cohort (*N* = 305,570). The propensity scores were generated by the above multivariable logistic regression model of baseline insulin as the dependent variable. The distribution of propensity scores was checked to ensure overlap between insulin use groups. We then used a Stata module (psmatch2) [21] to perform 1–1 matching by caliper without replacement on the estimated propensity scores between insulin use groups. Standardized mean differences in the covariates between the insulin use groups were evaluated before and after matching by examining a plot of the standardized mean differences across the covariates. There were no significant departures from balance following the application of propensity-score matching (maximum standardized difference = 4%).

In additional multivariable Cox regression analyses, we examined the joint associations of baseline insulin use and eGFR groups with serious hypoglycemic events using eGFR ≥90 and not on insulin as the reference group in the entire cohort (*N* = 855,133) and propensity score matched cohort (*N* = 305,570).

Statistical analysis was performed using Stata Version 15.1 (College Station, TX, USA).

## Results

Of the 855,133 veterans that met the criteria for the current analysis, 653,200 (76.4%) were not on insulin at baseline. In general, those on insulin had longer duration of diabetes, higher A1C, higher BMI and lower use of metformin and sulfonylureas (Table 1). They also had higher comorbidity burden with higher prevalence of history of heart failure, coronary artery disease and stroke. The baseline eGFR was lower in those on insulin compared to those not on insulin (69 ± 25 versus 74 ± 21 ml/min/1.73 m^2^).

**Table 1:** Baseline characteristics of veterans with T2D by baseline use in the entire analytic cohort (*N* = 855,133) and propensity score matched cohort (*N* = 305,570)

	Entire analytic cohort		Propensity score matched	
	On insulin	Not on insulin	On insulin	Not on insulin
	*N* = 201,933	*N* = 653,200	*N* = 152,785	*N* = 152,785
**Demographics**				
Age, (years)	65.1 ± 10.4	66.4 ± 11.0	65.4 ± 10.6	65.8 ± 10.9
Male, (%)	97	97	97	97
African American, (%)	21	17	20	20
**Comorbid Conditions**				
Congestive heart failure, (%)	16	8	12	12
Coronary artery disease, (%)	42	32	38	38
Myocardial infarction, (%)	6	4	5	5
Peripheral vascular disease, (%)	17	11	15	14
Stroke, (%)	14	11	13	12
Lung disease, (%)	24	21	22	22
Cancer, (%)	12	13	12	12
**Clinical Features**				
Systolic BP, (mmHg)	137 ± 21	136 ± 19	137 ± 20	137 ± 20
Diastolic BP, (mmHg)	75 ± 13	76 ± 12	75 ± 13	75 ± 13
Body mass index (kg/m^2^)	33 ± 7	32 ± 6	33 ± 7	33 ± 7
Estimated GFR, (ml/min/1.73 m^2^)	69 ± 25	74 ± 21	71 ± 24	72 ± 24
CKD stages, (%)				
Stage 2	39	49	42	42
Stage 3a	18	16	17	17
Stage 3b	12	7	10	10
Stage 4/5	7	2	5	5
Hemoglobin A1c (%)	7.9 (7.0–9.3)	6.6 (6.1–7.4)	7.6 (6.8–8.7)	7.4 (6.6–8.7)
Diabetes duration, (years)	6.8 (3.8–8.7)	3.8 (1.0–6.8)	6.1 (2.9–8.5)	6.0 (2.8–8.4)
**Medication Use**				
ACE inhibitor / ARB, (%)	78	64	75	75
Antilipemic agents (%)	79	70	78	71
Metformin (%)	44	48	48	49
Sulfonylurea, (%)	35	40	41	43
Thiazolidinediones, (%)	5	5	6	6

From the entire analytic cohort (*N* = 855,133), we derived propensity scores for baseline insulin use in a logistic regression model. In the propensity score matched cohort (*N* = 305,570), standardized % bias across covariates was close to zero (Supplemental Fig. 1) and baseline characteristics of those with (*N* = 152,785) and without insulin use (N = 152,785) at baseline was well balanced with propensity score matching (Table 1**)**.

### Associations of levels of eGFR with prevalent and incident insulin use

**Fig. 2:**
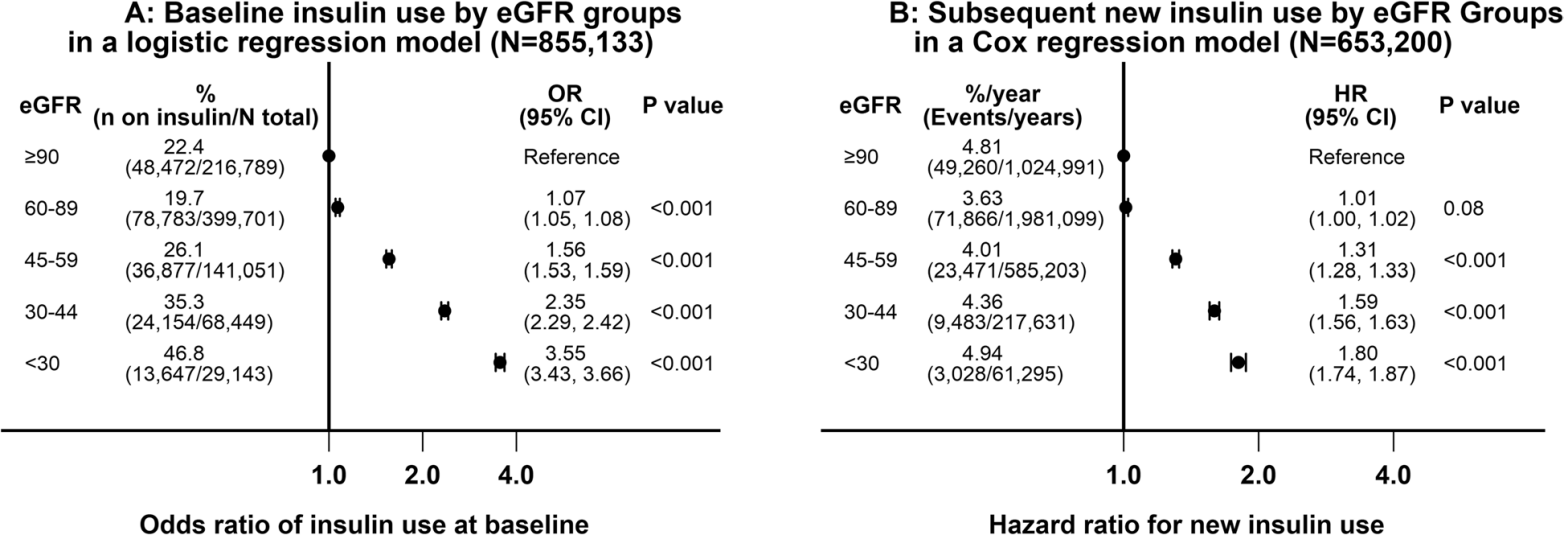
Associations of eGFR groups with baseline insulin use in the entire cohort in a logistic regression model (*N* = 855,133) and subsequent new insulin use in those without baseline insulin use (*N* = 653,200) in a Cox regression model

With adjustment for demographics, baseline comorbidities (listed in Table 1), blood pressures, BMI, duration of diabetes, HbA1C and medications, in a multivariable logistic regression model, compared to veterans with an eGFR of ≥90 ml/min/1.73 m^2^, a substantially higher percentage of veterans with stage 3A (odds ratio (OR) 1.56, 95% CI 1.53 to 1.59), stage 3B (OR 2.35, 95% CI 2.29 to 2.42) and stage 4/5 (OR 3.55, 95% CI 3.43 to 3.66) CKD used insulin at baseline (Fig. 2, Panel A).

In those without insulin use at baseline (*N* = 653,200), there were 157,108 persons who needed insulin over 3,870,219 years of follow-up with an event rate of 4.0/100-person years of follow-up. In a Cox regression model adjusted for the above covariates, compared to veterans with an eGFR of ≥90 ml/min/1.73 m^2^, veterans with stage 3A (hazards ratio (HR) 1.31, 95% CI 1.28 to 1.33), stage 3B (HR 1.59, 95% CI 1.56 to 1.63) and stage 4/5 (HR 1.80, 95% CI 1.74 to 1.87) CKD had substantially higher new insulin use during follow-up (Fig. 2, Panel B).

### Associations of baseline insulin use and levels of eGFR with subsequent serious hypoglycemic episodes

**Fig. 3:**
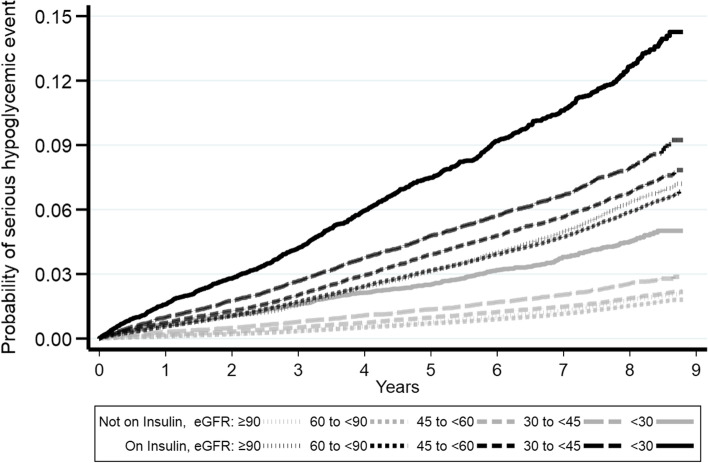
Kaplan-Meier curves for probability of serious hypoglycemic event by insulin and eGFR groups

The incidence of serious hypoglycemic events in veterans with T2D not on insulin (0.21%, 9435 events, 4,460,465 years of follow-up) was lower compared to those on insulin (0.84%, 10,558 events 1,257,904 years of follow-up). Kaplan-Meier curves showed that these differences persisted across different stages of CKD (Fig. 3).

**Fig. 4:**
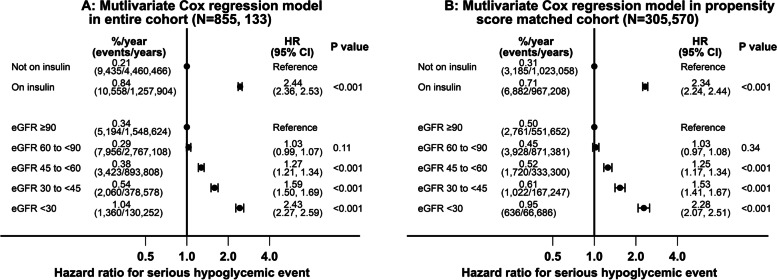
Individual associations of baseline insulin use and eGFR groups with serious hypoglycemic events in the entire cohort (*N* = 855,133) and propensity score matched cohort (*N* = 305,570)

In the entire analytic cohort (*N* = 855,133), in a multivariable Cox regression model adjusted for demographics, duration of diabetes, comorbidities, blood pressures, BMI, HbA1C, diabetes medications and use of ACE-I/ ARB, insulin use (HR 2.44, 95% CI 2.36 to 2.53) and more advanced CKD (HR 2.43, 95% CI 2.27 to 2.59 for comparison of eGFR < 30 versus ≥90 ml/min/1.73 m^2^ group) were both associated with increased risk of serious hypoglycemic events (Fig. 4, panel A). In a multivariable Cox regression model in the propensity score matched cohort (*N* = 305,570), the corresponding hazard ratios for hypoglycemic events for insulin use (HR 2.34, 95% CI 2.24 to 2.44) and more advanced CKD (HR2.28, 95% 2.07 to 2.51) were similar to that observed in the entire cohort (Fig. 4, panel B).

**Fig. 5:**
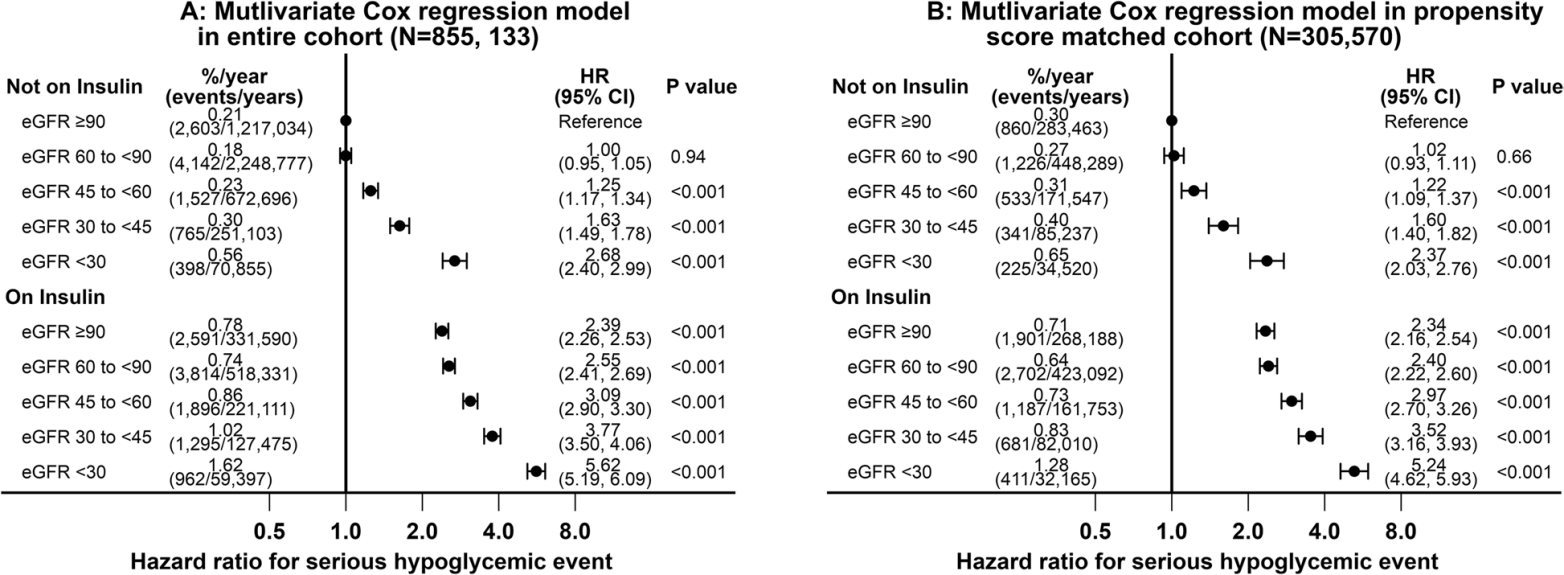
Joint associations of baseline insulin use and eGFR groups with serious hypoglycemic events using eGFR ≥90 and not on insulin as the reference group in the entire cohort (*N* = 855,133) and propensity score matched cohort (*N* = 305,570)

Using those not on insulin with eGFR ≥90 ml/min/1.73 m^2^ as the reference group, those on insulin with eGFR < 30 ml/min/1.73 m^2^ had the highest risk of hypoglycemia (in the entire analytic cohort HR 5.62, 95% CI 5.19 to 6.09 and in the propensity score matched cohort HR 5.24, 95% CI 4.62 to 5.93) with the other groups having intermediate risks (Fig. 5, panels A and B).

## Discussion

The main findings of this observational study are that insulin use was higher in more advanced CKD and that both insulin use and advanced CKD were independent risk factors for serious hypoglycemic events. Furthermore, compared to those with preserved kidney function and not on insulin, the risk of serious hypoglycemic events was nearly 5.3-fold higher in patients who used insulin and had eGFR < 30 ml/min/1.73 m^2^.

Previous literature suggests that because the kidney is responsible for the majority of exogenous insulin clearance, patients with diabetes and CKD and lower renal clearance rates have higher levels of serum insulin and may require less insulin than those without CKD [4, 5]. In contrast to this commonly held belief, the results of this study suggest that the need for insulin for glycemic control is inversely related to kidney function with a graded increase in baseline and subsequent insulin use with higher stages of CKD.

There are potential biological explanations for this observed finding. First, insulin resistance is common in CKD [22, 23], possibly related to pro-inflammatory cytokines such as interleukin-6 and tumor necrosis factor-⍺ [24] and oxidative stress [25–27] that are involved in intracellular mechanisms of insulin resistance [28–33]. Second, pancreatic beta-islet cells have low expression of antioxidant enzymes and because of this low antioxidant capacity, they are highly sensitive to oxidative stress [34–36]. Experimental data suggest that beta cell dysfunction might be worsened in CKD due to increased oxidative stress from the accumulation of uremic toxins [37]. Third, many of the anti-diabetic medications are contraindicated in advanced CKD [38–40]. Thus, this combination of decreased insulin production, peripheral insulin resistance and contraindications for other medications could increase the need for exogenous insulin for glycemic control in CKD.

There are also potential biological explanations for the associations of advanced CKD with higher risk of hypoglycemia. Renal gluconeogenesis plays an important role in countering hypoglycemia in healthy adults [41–43]. People with moderate to severe CKD have reduced kidney mass and therefore, a reduced capacity for glucose release from the kidneys [44] which might increase the risk for hypoglycemia. However, the previous data on whether CKD is a risk factor for hypoglycemia has been conflicting. Some of the previous studies noted such an association [45, 46] but not all [9].

We found not only that the need for insulin was higher in more advanced CKD but that CKD and insulin use are independent factors that contribute to the risk of a hypoglycemic event and that this risk was the highest in patients with advanced CKD on insulin. This finding is of clinical significance for therapeutic options for glycemic control in advanced CKD as hypoglycemia has been associated with higher risk of mortality, cardiovascular disease, cognitive impairment and progression of CKD [47–50]. A previous study noted that in patients hospitalized due to an acute kidney injury, hypoglycemic events were most common in insulin users [20]. The safety profile of insulin compared to newer glycemic agents such as SGLT-2 inhibitors and GLP-1 analogs in advanced CKD need to be further examined in randomized controlled trials to determine optimal glycemic control therapy in this population.

The major limitation of the current study is the observational nature of the analyses despite the use of propensity score matching as there may be residual confounding from unknown covariates that were not included in the development of propensity scores. While the definitions of hypoglycemia relied on data from electronic medical records is also a limitation, we used a previously validated definition [19, 20]. Even though, we used a large national database of veterans, it is possible that serious hypoglycemic events that were treated at non-VA medical were not captured. However, such underreporting will likely bias the study towards the null hypothesis and therefore, the currently reported results are likely conservative estimates of the associations of insulin use and advanced CKD with the risk of hypoglycemic events. Lastly, the study cohort was predominantly male (97%); future studies of hypoglycemia in both women and men are warranted.

## Conclusions

In summary, contrary to widely held assumption that advanced CKD is associated with decreased need for insulin, we found that insulin use was greater in T2D patients with more advanced CKD. Furthermore, this study also found that both insulin use and CKD are independent factors for risk of hypoglycemia, with patients with advanced CKD who use insulin being at the highest risk for a hypoglycemic event. Future randomized controlled trials are needed to determine the safety of insulin compared to newer glycemic agents in patients with T2D and advanced CKD.

## Supplementary Information

**Additional file 1: Supplemental Table 1.** ICD-9 codes used to define medical conditions. **Supplemental Table 2.** ICD-9/10 codes used to define hypoglycemic events. **Supplemental Figure 1.** Standardized % bias across covariates by baseline insulin use before and after propensity score matching.

## Data Availability

The data that support the findings of this study are available from Veterans Affairs, but restrictions apply to the availability of these data, which were used under license for the current study, and so are not publicly available.

## Abbreviations

DM: Diabetes mellitus
CKD: Chronic kidney disease
T2D: Type 2 diabetes
eGFR: Estimated glomerular filtration rate
VA: Veterans Affairs
BMI: Body mass index
HbA1C: Hemoglobin A1C
VINCI: VA Informatics and Computing Infrastructure
CDW: VA Corporate Data Warehouse
ICD: International Classification of Disease
TZD: Thiazolidinedione
ACEI: Angiotensin converting enzyme inhibitors
ARB: Angiotensin receptor blocker
