# Supplementary material for: Insulin use in chronic kidney disease and the risk of hypoglycemic events
Source: BMC Nephrol. 2022 Feb 21;23:73. doi: 10.1186/s12882-022-02687-w (PMC8862360; doi:10.1186/s12882-022-02687-w)
Supplement: Supplementary file 1 — Additional file 1: Supplemental Table 1. ICD-9 codes used to define medical conditions. Supplemental Table 2. ICD-9/10 codes used to define hypoglycemic events. Supplemental Figure 1. Standardized % bias across covariates by baseline insulin use before and after propensity score matching. [file 12882_2022_2687_MOESM1_ESM.docx]

**Supplemental Material Table of Contents**

| **Supplemental Table 1** | ICD-9 codes used to define medical conditions | 2 |
| --- | --- | --- |
| **Supplemental Table 2** | ICD-9/10 codes used to define hypoglycemic events | 3 |
| **Supplemental Figure 1** | Standardized % bias across covariates by baseline insulin use before and after propensity score matching | 4 |

**Supplemental Table 1: ICD-9 codes used to define medical conditions**

| Type 2 diabetes | 250.X0, 250.X2 |
| --- | --- |
| Type 1 diabetes | 250.X1, 250.X3 |
| Congestive heart failure | 428.X, 398.91, 402.01, 402.11, 404.01, 404.03, 404.11, 404.13, 404.91, 404.93 |
| Coronary artery disease | 410.X, 411.X, 412.X, 413.X, 414.X, V45.81, V45.82 |
| Myocardial infarction | 410.X, 412.X |
| Peripheral vascular disease | 93.0, 437.3, 440.X, 441.X, 443.1-443.9, 47.1, 557.1, 557.9, V43.4 |
| Stroke | 362.34, 430.0-438.99 |
| Lung disease | 416.8, 416.9, 490.0-505.99, 506.4, 508.1, 508.8 |
| Cancer | 140.0-172.99, 174.0-195.8, 196.0-199.99, 200.0-208.99, 238.6 |

**Supplemental Table 2: ICD-9 codes used to define hypoglycemic events**

| Hospitalization | 251.0-251.2, 962.3, 270.3 |
| --- | --- |
| Emergency room visits | 250.3, 250.8, 251.0-251.2, 270.3, 962.3 |
| Exclusionary co-diagnoses if 250.8 is present | 259.8, 272.7, 681.X, 682.X, 686.9X, 707.1-707.9, 709.3, 730.0-730.2, 731.8 |

**
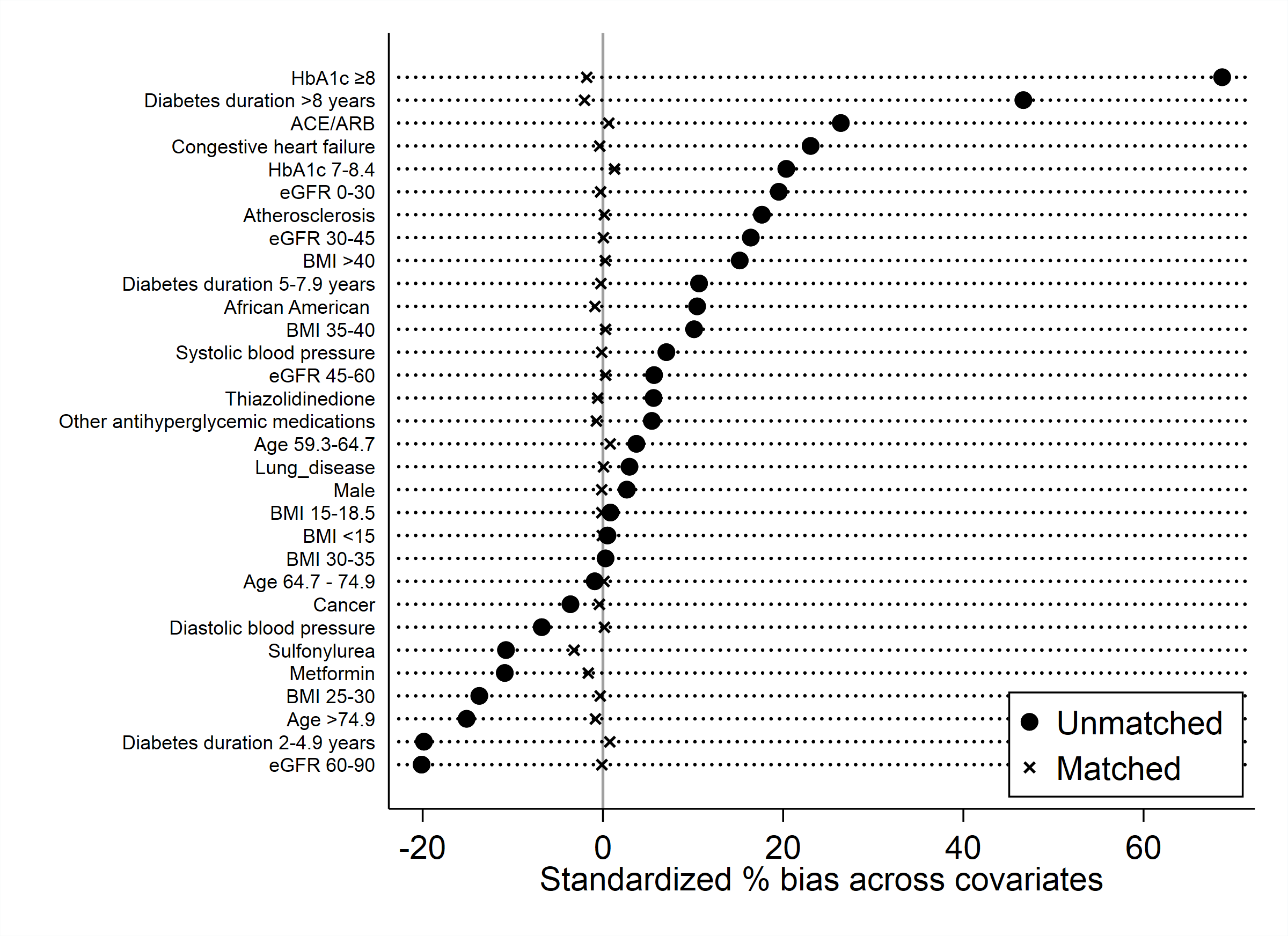
Supplemental Figure 1: Standardized % bias across covariates by baseline insulin use before and after propensity score matching**
